# *Buddleja officinalis Maximowicz* Extract Inhibits Lipid Accumulation on Adipocyte Differentiation in 3T3-L1 Cells and High-Fat Mice

**DOI:** 10.3390/molecules17078687

**Published:** 2012-07-23

**Authors:** Changhyun Roh, Min-Kyoung Park, Hee-June Shin, Uhee Jung, Jin-Kyu Kim

**Affiliations:** 1 Division for Biotechnology, Advanced Radiation Technology Institute (ARTI), Korea Atomic Energy Research Institute (KAERI), 1266, Sinjeong-dong, Jeongeup, Jeonbuk 580-185, Korea; Email: hj-shin@kaeri.re.kr (H.-J.S.); uhjung@kaeri.re.kr (U.J.); jkkim@kaeri.re.kr (J.-K.K.); 2 Department of Chemistry, Seoul National University, Gwanak-ro, Gwanak-gu, Seoul 151-747, Korea

**Keywords:** anti-obesity, lipid inhibition, adipocyte differentiation, *Buddleja officinalis Maximowicz* extract

## Abstract

Obesity is a global health problem. It is also known to be a risk factor for the development of metabolic disorders, type 2 diabetes, systemic hypertension, cardiovascular disease, dyslipidemia, and atherosclerosis. In this study, we elucidated that *Buddleja officinalis Maximowicz *extract significantly inhibited lipid accumulation during 3T3-L1 adipocyte differentiation. Furthermore, *Buddleja officinalis Maximowicz *extract reduced the body weight gain induced through feeding a high-fat diet to C57BL/6 mice. The treatment of *Buddleja officinalis Maximowicz *extract significantly reduced the adipose tissue weight to 2.7/100 g of body weight in high-fat mice. When their adipose tissue morphology was investigated for histochemical staining, the distribution of cell size in the high-fat diet groups was hypertrophied compared with those from *Buddleja officinalis Maximowicz *extract-treated mice. In addition, in *Buddleja officinalis Maximowicz *extract-treated mice, a significant reduction of serum triglyceride and T-cholesterol was observed at to 21% and 17%, respectively. The discovery of bioactive compounds from diet or dietary supplementation is one of possible ways to control obesity and to prevent or reduce the risks of various obesity-related diseases. These results support that *Buddleja officinalis Maximowicz* extract is expected to create the therapeutic interest with respect to the treatment of obesity.

## 1. Introduction

Obesity is a chronic metabolic disorder caused by an imbalance between energy intake and expenditure. Overweight and obesity are defined as abnormal or excessive fat accumulation presenting a health risk [1,2,3,4]. Many scientific communities have become increasingly interested in the molecular regulation of triglyceride synthesis and in pharmaceutical approaches to reduce fat absorption and storage due to phytochemicals, presenting an exciting opportunity for the discovery of new anti-obesity agents [5,6,7,8,9,10]. Globally, one in three of the world’s adults are overweight and one in ten is obese. The World Health Organization (WHO) estimates that the number of chubby adults will balloon to 2.3 billion (over 10% of the world’s population) by 2015, which is equal to the combined populations of China, Europe, and the U.S. In spite of many efforts to reduce body weight, the obese population has been continuously growing. While several types of therapeutics for obesity are currently available, but most of them have side effects [11,12,13,14]. Thus, new safer and more effective anti-obesity drugs are needed. During the last decade, researchers have focused on the discovery of new drugs to reduce obesity, which is one of the most important health issues of modern society. The discovery of bioactive compounds from diet or dietary supplementation is one possible way to control obesity and prevent or reduce the risks of getting various obesity-related diseases. Plants have been used as traditional natural medicines for healing many diseases. In particular, various oriental medicinal plants are reported to have biological activity [15]. Recently, there has been remarkable interest in finding natural lipid inhibitors from natural plants to replace synthetic compounds. Natural lipid inhibitor substances are presumed to be safe since they occur in plant foods, and are seen as more desirable than their synthetic substances. Scientific reports have shown that natural plants contain a large variety of substances that possess lipid inhibition activity. 

The flowering bud of *Buddleja officinalis Maximowicz* (Loganiaceae) is one of the earliest and most important crude herbs used for treatment in traditional Oriental medicine. *Buddleja officinalis Maximowicz *has been used traditionally as a folk medicine in Korea and China to treat vascular diseases, conjunctival congestion, clustered nebulae, and as an antiseptic [16]. *Buddleja officinalis Maximowicz *has been reported to contain terpenoids, flavonoids, phenylethanoids, and saponins [17]. Although some constituents of *Buddleja officinalis Maximowicz *have been identified, there have been few studies on the pharmacological effects of a crude extract for proving its pharmacological activities. In this study, we elucidated the lipid inhibition activity of *Buddleja officinalis Maximowicz* extract from *in vitro* and *in vivo* activities. To the best of our knowledge, this is the first report showing that *Buddleja officinalis Maximowicz* extract demonstrates lipid inhibition activity. 

## 2. Results and Discussion

### 2.1. Effect of Lipid Accumulation and Cell Viability in 3T3-L1

**Figure 1:**
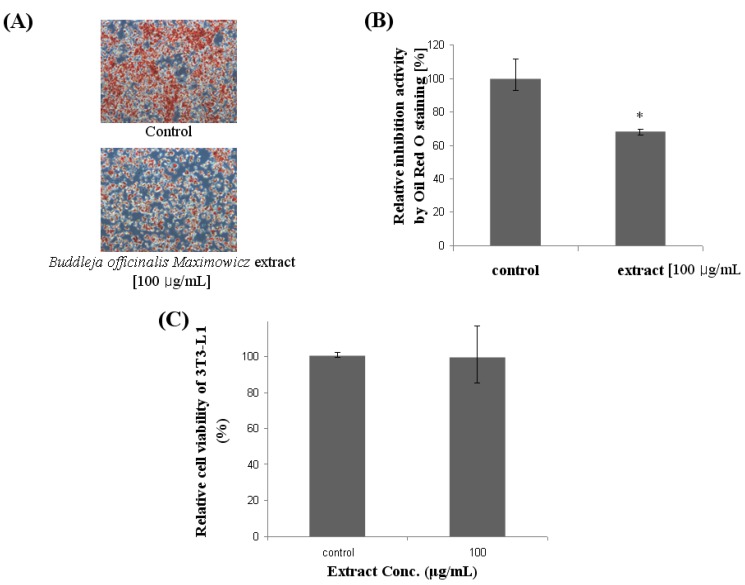
Effect of *Buddleja officinalis Maximowicz* extract on Oil Red O staining in cultured 3T3-L1 adipocytes. (**A**) Effect of *Buddleja officinalis Maximowicz *extract on fat droplet formation in 3T3-L1 cells. Fat droplets in preadipocytes were differentiated with 100 μg/mL of *Buddleja officinalis Maximowicz* extract treatment for 10 days and it was stained with Oil Red O dye and examined using a light microscope; (**B**) Relative inhibition by quantification method of Oil Red O staining. Values are expressed as mean ± standard deviation of at least three independent from that of the control treatment. Values are means ± SE (n = 3), ** *p* < 0.001; (**C**) Cell viability by MTT assay.

As shown in Figure 1, *Buddleja officinalis Maximowicz *extract at 100 μg/mL attenuated lipid accumulation in differentiated adipocytes as evidenced by Oil Red O staining. *Buddleja officinalis Maximowicz *extract was found to significantly reduce lipid accumulation in 3T3-L1 adipocytes, suggesting anti-obesity activity. In Figure 1A,B, the effect of *Buddleja officinalis Maximowicz *extract on fat droplet formation in 3T3-L1 cells, and relative inhibition through the quantification method of Oil Red O staining, was demonstrated. In presence of 100 μg/mL treatment with the extract lipid accumulation in differentiating 3T3-L1 cells was significantly reduced by 32%. 3T3-L1 preadipocytes were differentiated in the presence of *Buddleja officinalis Maximowicz *extract for 6 days. When the cytotoxicity of *Buddleja officinalis Maximowicz *extract was examined using an MTT assay, *Buddleja officinalis Maximowicz *extract in concentrations of 100 μg/mL showed no significant toxicity of preadipocytes. The cell viability remained about 90~100% in the nontoxic range (Figure 1C).

### 2.2. Mice Characteristics and White Adipose Tissue

**Figure 2:**
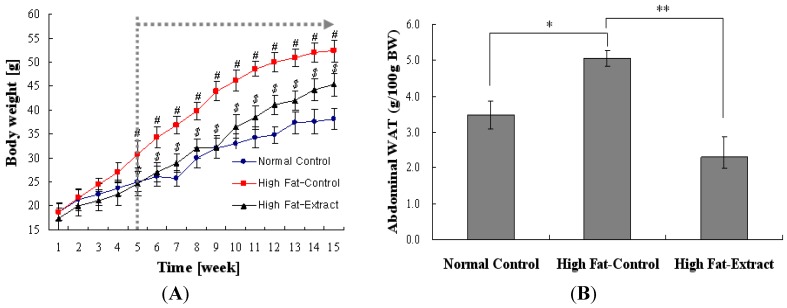
Effect of *Buddleja officinalis Maximowicz* supplementation on body weight in C57BL/6 fed high-fat diet. Values are means ± SE (n = 9 per group) #, *p* < 0.05 *vs.* normal diet. $ *p* < 0.05, high fat-extract *vs*. high-fat diet (**A**) and abdominal white adipose tissue (gWAT) in organ. Values are means ± SE (n = 9 per group) * *p* < 0.05 *vs*. normal diet; ** *p* < 0.001 high fat-extract *vs*. high fat diet (**B**).

The composition of the diets is presented in Table 1. It was compared to the fat ratio of diets in Experimental 3.4. Figure 2A represents the changes in body weight of the groups during the experiments. Feeding a high-fat diet containing Lard caused a marked increase in body weight as compared to a normal diet. However, feeding a high-fat diet plus *Buddleja officinalis Maximowicz *extract at levels of 200 mg/kg body weight significantly reduced the body weight gain induced by the high-fat diet (Figure 2A). Food intake during the experiments was weighed. Feeding a high-fat diet caused a marked decrease in food intake as compared to feeding a normal diet, but *Buddleja officinalis Maximowicz *extract did not affect food intake (data not shown).

**Table 1:** Composition of the diets.

Composition (g/kg)	Normal	High-Fat
Casein	200	200
L-Cystine	3	3
Corn starch	315	0
Maltodextrin 10	35	125
Sucrose	350	68.8
Cellulose, BW200	50	50
Soybean oil	25	25
Lard *	20	245
Mineral Mix S10026	10	10
Dicalcium phosphate	13	13
Calcium carbonate	5.5	5.5
Potassium citrate	16.5	16.5
Choline bitartrate	2	2

* *p* < 0.05 *vs*. normal diet.

**Figure 3:**
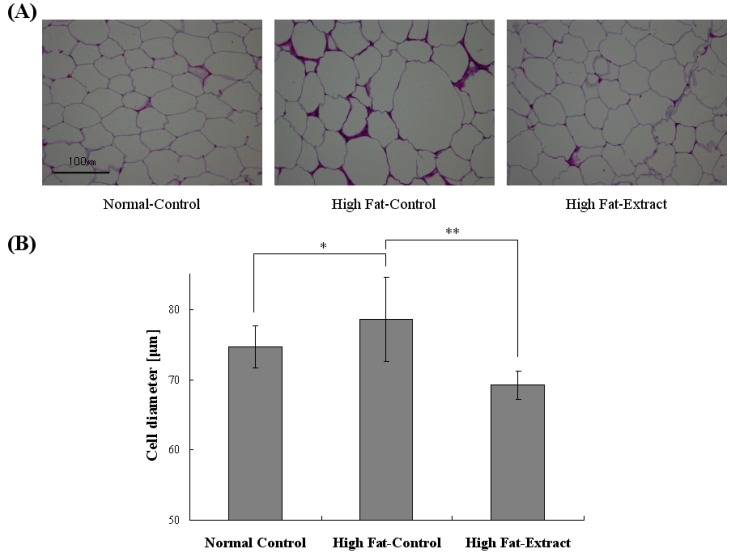
Effect of *Buddleja officinalis Maximowicz* extract on abdominal white adipose tissue (WAT) in high-fat diet mice (**A**) Histological analysis of WAT from control and high-fat diet and *Buddleja officinalis Maximowicz* treated high-fat model (**B**) Average fat cell size. Values are means ± (n = 9 per group) * *p* < 0.05 *vs*. normal diet; ** *p* < 0.001 high fat extract *vs.* high fat diet.

In Figure 2B, the effects of *Buddleja officinalis Maximowicz *extract on abdominal white adipose tissue weight in mice fed a high-fat diet for 15 weeks are shown. The mice fed a high-fat diet containing lard for 15 weeks have a significantly higher adipose tissue weight than mice fed a normal diet. The treatment of *Buddleja officinalis Maximowicz *extract significantly reduced the adipose tissue weight to 2.7/100 g body weight in high-fat mice. When the adipose tissue morphology was investigated for histochemical staining, the distribution of cell size in the high-fat diet groups was hypertrophied compared with those from *Buddleja officinalis Maximowicz *extract-treated mice (Figure 3A). In Figure 3B, the quantification of adipocyte size confirmed the distribution of cell size with mean diameters decreasing from 78 ± 6 μm in the high-fat diet groups, and to 69 ± 2 μm in the *Buddleja officinalis Maximowicz *extract-treated groups. Feeding the high-fat diet induced hypertrophy of the adipocytes in abdominal WAT compared with that of the normal diet (Figure 3A,B). The hypertrophy did not occur in the high fat + *Buddleja officinalis Maximowicz *extract group (Figure 3A,B). The results obtained from the other mice were similar to those shown in Figure 3. This result showed a significant difference among groups which means the findings that *Buddleja officinalis Maximowicz *extract can prevent the lipid accumulation.

### 2.3. Biochemical Study

**Figure 4:**
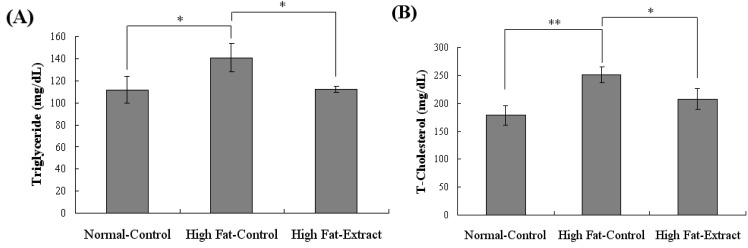
Effects of *Buddleja officinalis Maximowicz* treated on body weight in C57BL/6 fed high-fat diet (**A**) Triglyceride. Values are means ± SE (n = 9 per group) * *p* < 0.05 *vs.* normal diet; * *p* < 0.05 high fat-extract *vs*. high fat diet (**B**) T-Cholesterol. Values are means ± SE (n = 9 per group) ** *p* < 0.001 *vs*. normal diet; * *p* < 0.05 high fat-extract *vs.* high fat diet.

The serum triglyceride and T-cholesterol levels were higher in the high-fat diet alone group than in the normal diet group. In Figure 4, a significant reduction of serum triglyceride and T-cholesterol was observed at to 21% and 17%, respectively, in *Buddleja officinalis Maximowicz* extract-treated mice. The present study suggests that a promising anti-obesity agent like *Buddleja officinalis Maximowicz *extract might be of therapeutic interest for the treatment of obesity. The novelty of this study is in that we clarified the relationship between the level of *Buddleja officinalis Maximowicz* extract to the inhibition of lipid accumulation on adipocyte differentiation in 3T3-L1 cells and its effect on body fat under the influence of a high-fat diet. In addition, *Buddleja officinalis Maximowicz* extract showed T-cholesterol and triglyceride lowering effects with the same diet. However, further work is needed to elucidate the possible mechanisms by which *Buddleja officinalis Maximowicz *extract intervenes.

## 3. Experimental

### 3.1. Materials and Preparation of Natural Plant Extracts

High-glucose DMEM, bovine calf serum (BCS), and fetal bovine serum (FBS) were purchased from Hyclone (Logan, UT, USA). Trypsin/EDTA, insulin, Dexamethasone, and 3-isobutyl-1-methylxanthine (IBMX) were purchased from Sigma-Aldrich (St. Louis, MO, USA). All reagents were of the highest grade available. *Buddleja officinalis Maximowicz* was purchased from Deokhyeon-dang (Kyungdong market, Seoul, Korea) and authenticated by Dr. H.K. Lee, Korea Research Institute of Bioscience & Biotechnology (KRIBB). The specimen of *Buddleja officinalis Maximowicz* has been deposited at the Plant Extract Bank in KRIBB. The dried and powdered plant was extracted three times with distilled water by heating at 100 °C for 1 h, and the extract was obtained through the removal of the solvent during evaporation. It was then freeze-dried to obtain the extracts. The samples were stored at −20 °C for further study.

### 3.2. Cell Culture and Differentiation

3T3-L1 preadipocytes were obtained from ATCC (Manassas, VA, USA). 3T3-L1 preadipocytes were grown in DMEM supplemented with 10% (v/v) heat-inactivated FBS at 37 °C in an atmosphere containing 5% CO2. To induce adipocyte differentiation, 2-day post-confluent 3T3-L1 preadipocytes (day 0) were stimulated for 48 h (day 2) with an inducer (10 μg/mL insulin, 2.5 μM dexamethasone, and 0.5 mM 3-isobutyl-1-methylxanthine) including natural extracts, and then maintained for 6 days (day 8) in DMEM supplemented with 10% FBS and 10 μg/mL insulin including natural extracts. 3T3-L1 cells were treated with natural extracts in DMEM supplemented with 10% FBS for 2 days (day 10). To examine the effect of natural extracts on adipocyte differentiation in 3T3-L1 cells, the media and natural extracts were changed every 2 days until the end of the experiment at day 10. 

### 3.3. Cell viability and Oil Red O Staining Intracellular Triglycerides

Cell cytotoxicity was determined colorimetrically using an MTT assay [18]. Cells cultured in the DMEM medium were treated with natural extracts at a final concentration of 100 μg/mL every 2 days, and then treated with a 5 mg/mL MTT (3-(4,5-dimethyl-2-thiazolyl)-2, 5-diphenyltetrazolium bromide) solution (Sigma) for 3 h. After the cells were dissolved in 0.04 N HCl (in isopropanol), the formazane level was analyzed by measuring the OD 570 nm (against OD 630 nm) [19]. 3T3-L1 adipocytes were washed with PBS and fixed with 10% formalin for 30 min. After two washes with distilled water, the cells were stained for at least 1h at room temperature in a freshly diluted Oil Red O solution (0.5% Oil Red O in isopropanol as a stock solution). Finally, the dye retained in the 3T3-L1 cells was eluted with isopropanol and quantified by measuring the optical absorbance at 500 nm.

### 3.4. Mice and Diet

C57BL/6 female mice were used in the present study. Young mice (6 weeks of age) were purchased from Orient Bio Co., Ltd. (Charles River Technology, Songham, Korea). The mice were housed 5–10 per cage under specific pathogen-free conditions at 23 °C ± 3 °C and 50 ± 10% relative humidity, and were provided with nutritional chow 5L79 (PMI Nutrition LLC, St Louis, MO, USA). The mice were given free access to water and were fed on a standard diet and a high-fat diet for 15 weeks. The HF diet was prepared by mixing the ingredients as listed in Table 1 [20]. The resulting nutrient composition was as follows: the HF diet contained 26.2% protein, 34.9% fat, 26.3% carbohydrate, 3.6% ash, 1% fiber, and 8.0% moisture; the normal diet contained 19.2% protein, 4.3% fat, 67.3% carbohydrate, 3.6% ash, 1% fiber, and 4.6% moisture. The unit calorie for HF diet was 5.24 Kcal/g, for normal diet 3.85 Kcal/g. The chows were kept in −20 °C until being used. The experimental group was orally administrated with *Buddleja officinalis Maximowicz* extract at a concentration of 200 mg/kg body weight for 15 weeks. The composition of diets and fat ratio are shown in Table 1. These experiments were approved by the Korea Laboratory Animal Care and Use Committee. All procedures were conducted in accordance with the ethical guidelines in the ‘Animal Care Act’, prepared by the Ministry of Agriculture and Forestry, Korea. 

### 3.5. Adipose Tissue Morphology and Biochemical Assay

For hematoxylin and eosin (H&E) staining, the samples were fixed in 10% formalin, prepared as paraffin blocks, sectioned at 6 μm, and stained with H&E. The prepared specimen was examined using a light microscope at 100× magnification. After sacrifice, the abdominal WAT was weighed between groups. To measure the biochemical analysis in serum, the animals were deprived of food for 15 h, and blood samples were then collected from the veins of the mice under carbon dioxide anesthesia. Triglyceride concentration was measured enzymatically using commercial kits and following the manufacturer’s procedures (ASAN Pharm. Co., Seoul, Korea). T-cholesterol in serum was measured through an enzymatic colorimetric method using a commercial kit (ASAN Pharm. Co., Seoul, Korea).

### 3.6. Statistics

A statistical analysis was performed using a one-way analysis of variance (ANOVA), and inter group comparisons were made using Tukey’s Multiple Comparison test. The values are expressed as average ± standard deviation. The *p* value as 0.05 or 0.001 was considered as significant.

## 4. Conclusions

In conclusion, we have demonstrated that *Buddleja officinalis Maximowicz* extract had anti-obesity activity by reducing the lipid accumulation in 3T3-L1 cells. Furthermore, the extract reduced the body fat mass and body fat percentage in C57BL/6 mice fed a high-fat diet. In addition, *Buddleja officinalis Maximowicz *extract significantly showed triglyceride and T-cholesterol-lowering effects with the same diet. These results suggest that *Buddleja officinalis Maximowicz* extract ought to be considered as a useful anti-obesity food. More experiments, including in humans, are required. In light of such beneficial effects, *Buddleja officinalis Maximowicz* extract has potential use as a functional nutrient for preventing lifestyle-related obesity diseases in humans who tend to ingest a diet containing a high level of fat.
